# Detraining Effects Following Chronic Stretching Training on Range of Motion: A Systematic Review and Meta-Analysis

**DOI:** 10.1186/s40798-025-00935-5

**Published:** 2025-11-21

**Authors:** Andreas Konrad, Konstantin Warneke, Olyvia Donti, Ioli Panidi, Vasiliki Gaspari, Masatoshi Nakamura, Ewan Thomas, Gregory C. Bogdanis

**Affiliations:** 1 Institute of Human Movement Science, Sport and Health, University of Graz, Mozartgasse 14, Graz, 8010 Austria; 2 School of Physical Education and Sport Science, National and Kapodistrian University of Athens, Athens, 17237 Greece; 3 Faculty of Rehabilitation Sciences, NishiKyushu University, 4490-9 Ozaki, Kanzaki, 842-8585 Saga Japan; 4 Sport and Exercise Sciences Research Unit, Department of Psychology, Educational Science and Human Movement, University of Palermo, Via Giovanni Pascoli 6, Palermo, 90144 Italy

**Keywords:** Stretch training, Long-term stretching, Training cessation

## Abstract

**Background:**

Stretch training can chronically increase the range of motion (ROM) of a joint. However, to date, there is still a need for a comprehensive synthesis of knowledge regarding the effects of training cessation (i.e., detraining) on ROM gains from stretching.

**Objectives:**

The purpose of this systematic review with meta-analysis was to investigate the detraining effects of stretch training on ROM.

**Methods:**

PubMed, Scopus, and Web of Science were searched. Controlled trials and randomized controlled trials with a training and a detraining phase that involved any type of stretching training, with healthy participants of all ages, were included. Risk of bias was assessed using Cochrane RoB2 and ROBINS-I tools. A meta-analysis was performed to assess effect sizes for ROM changes between pre-intervention to post-intervention, pre-intervention to detraining, and post-intervention to detraining. Additionally, the effects of potential moderators were assessed (i.e., stretching technique, duration per session, frequency per week, duration of detraining, of the training period, and type of activity performed during detraining). GRADE analysis was used to determine the quality of evidence.

**Results:**

Out of the 189 records retrieved, 13 studies with a total of 556 participants were included in this review. These studies involved stretch training programs lasting from five to 15 weeks, followed by a detraining phase ranging from two to six weeks. Risk of bias was low in 74.3–77.1% of all criteria. Confidence in cumulative evidence was high for ROM gains and low for ROM maintenance following detraining. In the studies examined stretching training induced large increases in ROM (ES = 0.93, 95% CI: 0.54–1.31, *p* < 0.001). A moderate higher ROM was found after a detraining period compared to pre-intervention (ES = 0.55; 95% CI: 0.33–0.77, *p* < 0.001), while small ROM decreases were observed from post-intervention until the end of the detraining period (ES=-0.41; 95% CI: -0.73 – -0.09, *p* = 0.016). Moderator analysis showed that the frequency of stretching per week significantly moderated the ROM enhancements when comparing post-intervention to detraining only, and that only active participants maintained ROM from post-intervention to detraining, while other moderators showed no significant effects.

**Conclusions:**

Chronic gains in ROM are maintained above baseline levels after stretching training cessation. However, training cessation for 2–6 weeks slightly decreases ROM compared to post-intervention levels, indicating that to maintain ROM gains over time, some form of flexibility-enhancing method should be used.

**Supplementary Information:**

The online version contains supplementary material available at 10.1186/s40798-025-00935-5.

## Key Points

- Stretch training leads to large increases in range of motion in healthy participants, with an average effect size of 0.93, as evidenced by a systematic review and meta-analysis of 13 studies involving 556 participants.
- After the cessation of stretching training for 2–6 weeks (detraining period), there is a slight decrease in range of motion compared to the levels observed after the stretching intervention, as indicated by a small effect size of − 0.41. This suggests the importance of ongoing flexibility-enhancing methods to maintain ROM gains over time.
- After a period of detraining following stretch training, a moderate increase in range of motion was observed compared to the pre-intervention levels (ES = 0.55). This suggests that some residual benefits from the stretching training were retained even after the cessation of the training program.

**Supplementary Information:**

The online version contains supplementary material available at 10.1186/s40798-025-00935-5.

## Introduction

In various sports a high level of flexibility is essential [1]. In recent years, various studies have identified stretching as effective in improving flexibility and range of motion (ROM) in joints [2–4]. Notably, subgroup analyses of our latest meta-analysis have revealed that proprioceptive neuromuscular facilitation (PNF) stretching and static stretching lead to greater ROM increases compared to ballistic/dynamic stretching [2]. However, both PNF and static stretching are similarly effective in enhancing long-term ROM [2].

Despite this, currently the evidence regarding ROM maintenance following cessation requires overview. In sports training, a common approach is to periodize training [5], e.g., to focus on flexibility in the preparatory phase and to reduce or even stop flexibility training during the competitive period. Although some studies have examined ROM in the first days after stretch training [2], there is a gap in the literature regarding the impact on ROM several weeks after the stretch training has stopped, a period known as detraining [6, 7], deconditioning [7] or training cessation [6]. Indeed, understanding the impact of stopping stretch training on ROM might be crucial for athletes and coaches. Considering detraining in other physical components such as maximal oxygen uptake (VO2max), a meta-analysis [6] reported a significant reduction in VO2max in athletes. Regarding resistance training, detraining showed a detrimental effect on muscular performance (i.e., submaximal strength, maximal force, maximal power), while older people were shown to have a more pronounced decrease compared to younger people [8]. Existing studies on detraining following stretch training have yielded conflicting results, with some reporting increased ROM after detraining compared to the pre-intervention ROM [9, 10], while others observed no difference and hence, a decline in ROM back to baseline within the detraining phase [11, 12]. Moreover, some studies found that ROM after detraining was similar to post-intervention ROM [9, 13], while others reported a decline [11, 12]. Given these discrepancies, it is essential to consolidate the available evidence to gain a comprehensive understanding of detraining of stretching on ROM.

This can be achieved through randomized controlled trials or controlled trials that explore the impact of stretching training on ROM, incorporating a detraining phase. Therefore, this study aims to assess whether there is a reduction in ROM after several weeks without stretch training. Our investigation seeks to determine if ROM after detraining is maintained at a higher level compared with the post-intervention and the pre-intervention levels. Furthermore, potential moderating variables such as stretching technique, duration per session, frequency per week, duration of detraining or duration of the training period, and type of activity performed during detraining will be analyzed.

## Methods

This review was conducted according to the PRISMA guidelines and the suggestions from Moher et al. [14] for systematic reviews with meta-analysis. The review was registered in PROSPERO with the code CRD42024542884.

### Search Strategy

To identify all the relevant studies, we searched for eligible papers published until June 6th 2024. Similar to the previous studies [15, 16] the electronic literature search was performed in PubMed, Scopus, and Web of Science with the use of AND and OR Boolean operators. To find the eligible stretching studies the search term stretch* was used. Moreover, to assess studies on detraining effects the following search terms were included: detraining OR deconditioning OR “training cessation” OR “training reduction”. The systematic search was conducted by two independent researchers (AK, OD). Initially, the articles were screened by their title and then abstract. If the content remained unclear, the full text was retrieved for further screening and for identifying other relevant papers. Disagreements in the results were resolved by jointly reassessing the studies against the eligibility criteria. Once the authors agreed on the eligible papers from the database search, citations and references from those papers were checked as well.

### Inclusion and Exclusion Criteria

This review considered studies that investigated the chronic effects of stretching on ROM in healthy participants of all ages including a detraining phase, and all publication years were considered in the search. The studies were included when they were either randomized controlled trials or controlled trials with an intervention duration ≥ 2 weeks [17] as well as detraining duration ≥ 2 weeks. This implied that we excluded studies that examined the acute effects of stretching (or interventions shorter than < 2 weeks), investigated any combined treatment (e.g., stretching combined with strength training) or had another treatment as control condition, or did not consider a follow-up measure (or this being assessed before 2 weeks after training cessation). Moreover, we excluded review papers, case reports, special communications, letters to the editor, invited commentaries, conference papers, or theses.

### Data Processing and Statistics

Means (M) and standard deviations (SD) were extracted for pre-intervention values, post-intervention values and detraining values. In case of missing data, the authors of the primary studies were contacted. AK and OD double-checked the results.

We used the robust variance estimation (RVE) meta-analysis model to account for multiple, dependent study outcomes and heteroscedasticity of data distribution [18]. Standardized mean differences (SMD) and 95% confidence intervals (CI) were pooled between the intervention and control group for (1) pre-intervention to post-intervention, (2) pre-intervention to detraining (3) post-intervention to detraining. The between-study and intra-study heterogeneity was estimated using τ^2^ and Ω^2^, respectively. Pooled effect sizes (ES) were interpreted as follows: 0 ≤ ES < 0.2 trivial, 0.2 ≤ ES < 0.5 small, 0.5 ≤ ES < 0.8 moderate and ES ≥ 0.8 large [19]. The influence of potential moderators was evaluated using RVE meta-regression (for stretching frequency, duration and intervention period) and sensitivity analyses or subgroup analyses (for type of stretching, responder versus non-responders and activity level while in the detraining phase). All calculations were performed using R with the robumeta and the meta package [18].

### Risk of Bias Assessment and Methodological Quality

IP and OD independently assessed the risk of bias (RoB) of the included studies, and any conflict was resolved through discussion with AK and GCB. Risk of bias for randomized controlled trials and controlled trials without randomization was assessed using the updated Cochrane Risk of Bias 2 (RoB 2) and the Risk of Bias in Non-randomized Studies of Interventions (ROBINS-I), respectively. The sources of bias included in the updated Risk of Bias 2 (RoB2) Cochrane library were: bias arising from the randomization process, bias due to deviations from intended interventions (effect of assignment to intervention and effect of adhering to intervention), bias due to missing outcome data, bias in the measurement of the outcome, and bias in selection of the reported result [20] The sources of bias included in ROBINS-I were: bias due to confounding, bias in selection of participants into the study, bias in classification of interventions, bias due to deviations from intended interventions, bias due to missing data, bias in measurement of outcomes, and bias in selection of the reported results [20] Moreover, Egger’s regression intercept test and visual inspection of the funnel plots were applied to detect possible publication bias (considering independent effect sizes).

Quality and confidence in the cumulative evidence was evaluated following the “Grading of Recommendations, Assessment, Development and Evaluation” (GRADE) quality rating analysis [21]. In detail, limitations in study design or execution, inconsistency of results, indirectness of evidence, imprecision or publication bias each reduce one point until graded very low. On the contrary, for example, large magnitude effects or a dose-response gradient are criteria to upgrade the certainty level. As eight randomized controlled trials and five controlled trials were included in the meta study, initial quality was classified as moderate.

## Results

**Fig. 1:**
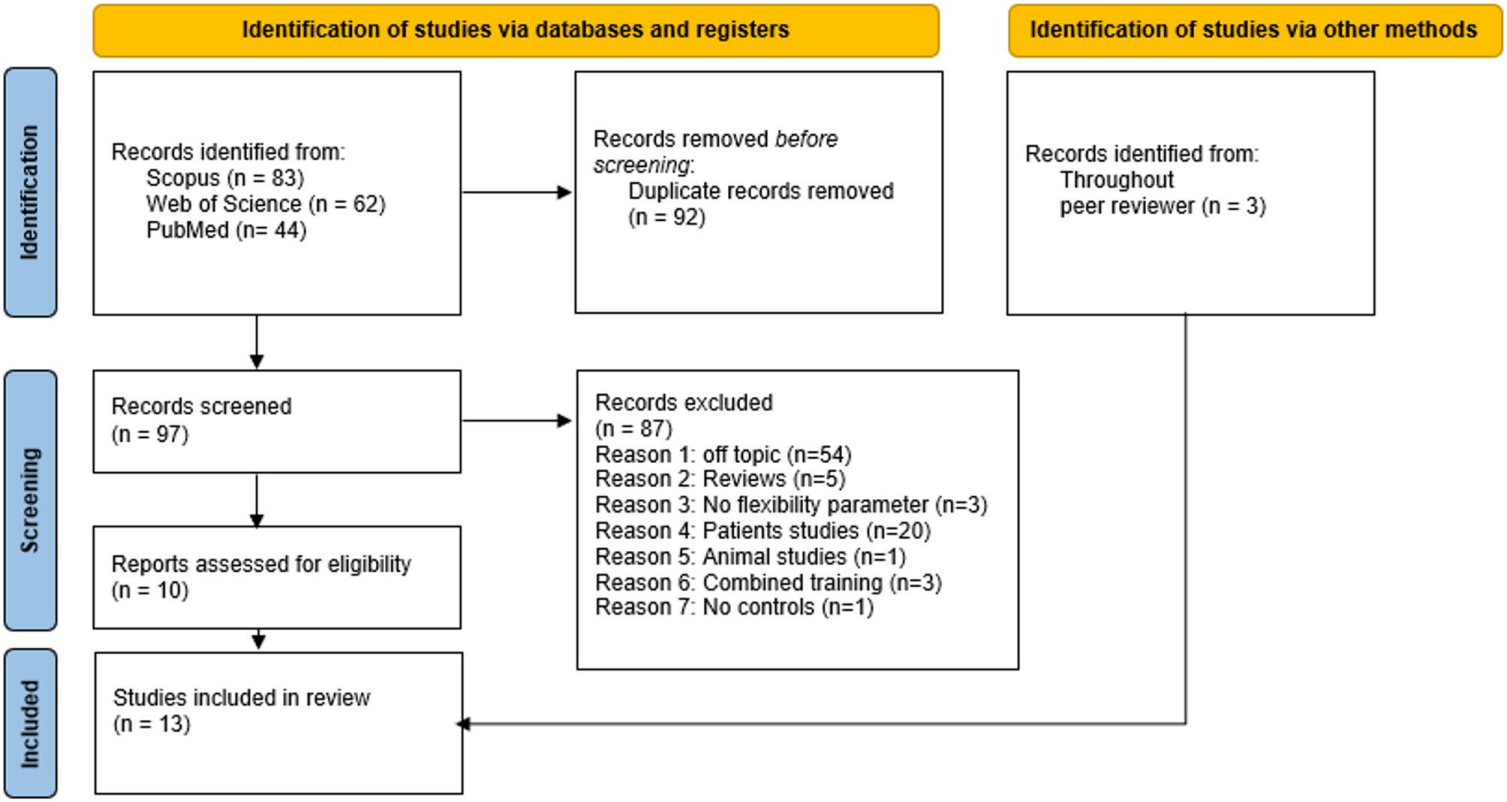
PRISMA flow-chart of literature search

Collectively, the search terms resulted in 189 records from the three databases. After title, abstract, and full text screening based on inclusion and exclusion criteria, a total of 10 eligible studies for the systematic review were identified. Additionally, throughout the further search process and peer review process 3 further studies were included. Those in total 13 eligible papers included 556 participants (176 m, 380 f; age: 19.9±15.5years with a range from 5.9 to 66 years) (see Fig. 1). These studies involved stretch training programs lasting from five to 15 weeks (average: 8.7 ± 3.2), followed by a detraining phase ranging from two to six weeks (4.3 ± 1.2). Table 1 shows the characteristics of individual studies.

**Table 1:** Study characteristics of the 13 eligible studies

Study	Stretch duration (weeks)	Stretching method	Muscle stretched	Frequency (stretching/week) and duration per session (sec)	Detraining duration (week)	Detraining activity	Participants characteristics	Outcome
Becerra-Fernández et al. [23]	8	Ballistic	Hamstrings	2/240	4	None	108 female adolescents pupils (age: 16.5)	Sit-and-reach
Bisconti et al. [40]	12	Static	Hip extensors, knee flexors	5/225	6	None	39 female and male (age: 23)	Knee ROM unilateral group, knee ROM bilateral group, ankle ROM unilateral group, ankle ROM bilateral group
Cipriani et al. [41]	4	Static	Hamstrings	3/60 6/60 7/60 14/60	4	None	53 female and male (age: 24)	Hip ROM - Daily stretching, twice each day; Hip ROM -Daily stretching, once each day; Hip ROM -Three-four days/week stretching, twice each day; Hip ROM -Three-four day/week stretching, once each day
Da Costa et al. [13]	12	Static	Knee extensors, knee flexors, dorsiflexors, plantar flexors	2/60	6	None	45 elderly female and male (age: 66)	Hip flexion ROM, Hip Extension ROM, Dorsiflexion ROM
Donti et al. [24]	15	Static	Hamstrings	3/90	2	Gymnastics	77 female gymnasts (age: 9)	Straight leg raise continuous group/leg, straight leg raise intermediate group/leg
Donti et al. [9]	9	Static	Quadriceps, hip flexor	3/90	3	Gymnastics	33 female gymnasts (age: 9.65)	Hip ROM unilateral-intermittent stretch (3 × 30 s), hip ROM unilateral-continuous stretch (90 s)
Guissard and Duchateau [22]	6	Static	Calves	5/600	4.28	None	12 female and male (age: 21 to 35)	Ankle ROM
Mayorga-Vega et al. [10]	8	Static	Hamstrings	2/300	5	None	45 female and male pupils (age: 10.9)	Sit-and-reach
Mayorga-Vega et al. [42]	8	Static	Hamstrings	2/300	5	None	45 female and male pupils (age: 9.89)	Sit-and-reach
Merino-Marban et al. [12]	8	Static	Hamstrings	2/60	5	None	45 female and male pupils (age: 5.91)	Sit-and-reach
Nakamura et al. [11]	5	Static	Calves	2/1800	5	None	15 sedentary males (age: 21.5)	Dorsiflexion ROM
Panidi et al. [25]	12	Static	Calves	5/540 to 900	3	Volleyball	21 female volleyball players (age: 13.5)	Ankle ROM
Willy et al. [43]	6	Static	Hamstrings	5/60	4	None	18 female and male (age: 21)	Knee Extension ROM

Sec = seconds; ROM = range of motion

### Risk of Bias Assessment

**Fig. 2:**
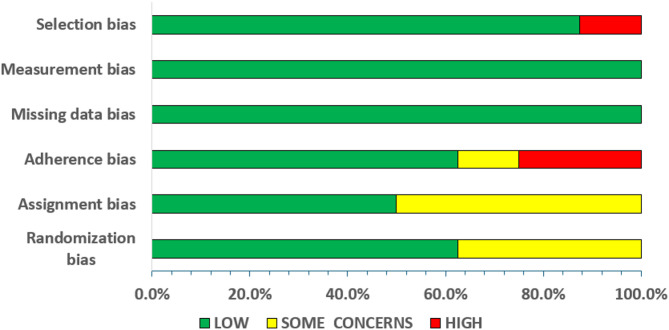
Summary of risk of bias assessment for randomized controlled trials

**Fig. 3:**
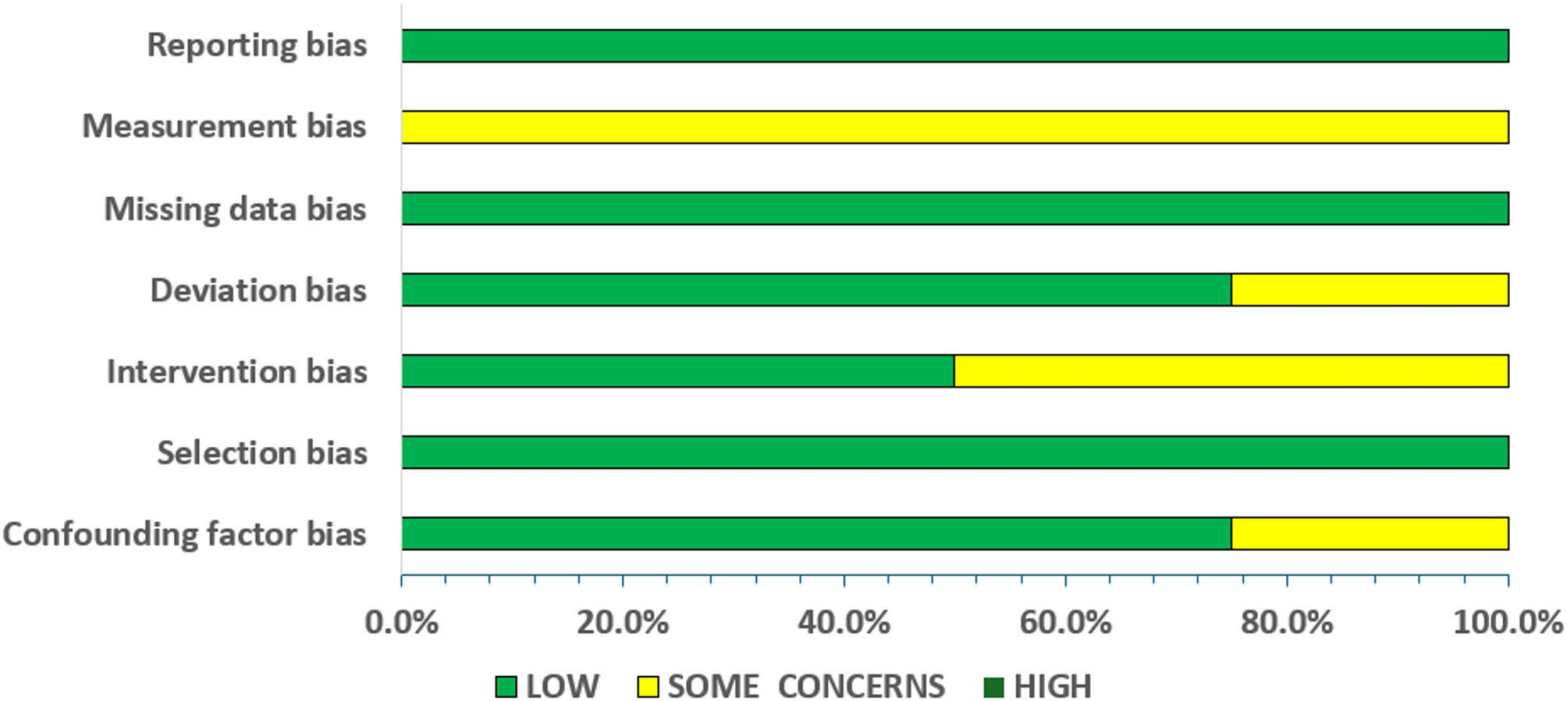
Summary of risk of bias assessment for controlled trials

A summary of the risk of the bias assessment is provided in Figs. 2 and 3 for the RCTs and CTs, respectively. For the RCTs and the CTs, risk of bias was low in 77.1 and 74.3% of all criteria, respectively. Detailed descriptions of the risk of the bias assessment for all the included studies are presented in the supplementary file 1 (S1) for the RCTs and CTs.

### Pre-Intervention to Post-Intervention Comparisons

**Fig. 4:**
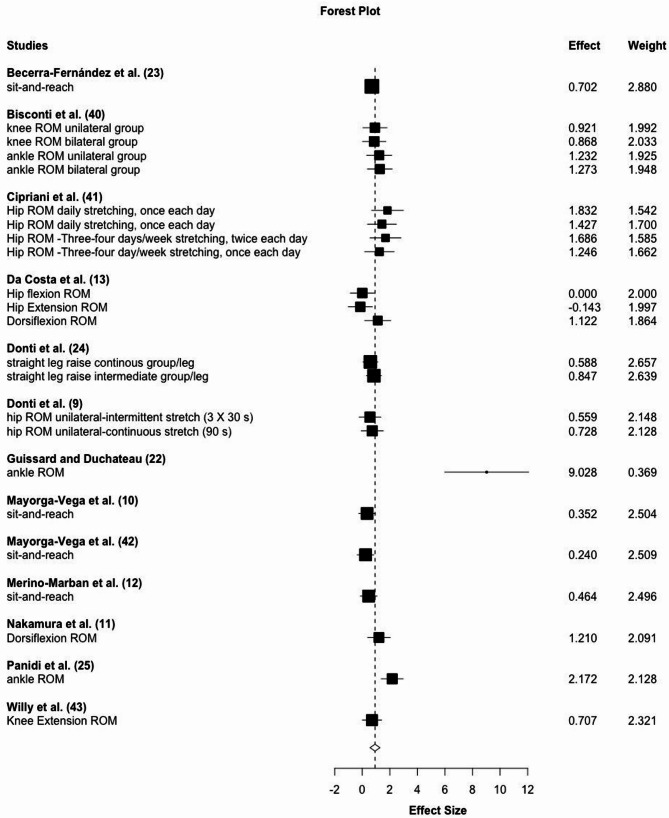
Forest plot for pre-intervention to post-intervention comparisons of ROM changes extracted from included studies

Results from 13 studies with 23 effects show large magnitude flexibility increases with ES = 0.93, 95% CI: 0.54–1.31, *p* < 0.001 without intra-study heterogeneity (Ω^2^ = 0) and small interstudy heterogeneity τ^2^ = 0.31 (See Fig. 4). Meta-regression was non-significant for stretching time per bout, stretching frequency per week or intervention period (number of weeks) (*p* = 0.08–0.49). All effects but one were calculated from static stretching. The sensitivity analysis for stretching types did not significantly affect the results.

### Pre-Intervention to Detraining Comparison

**Fig. 5:**
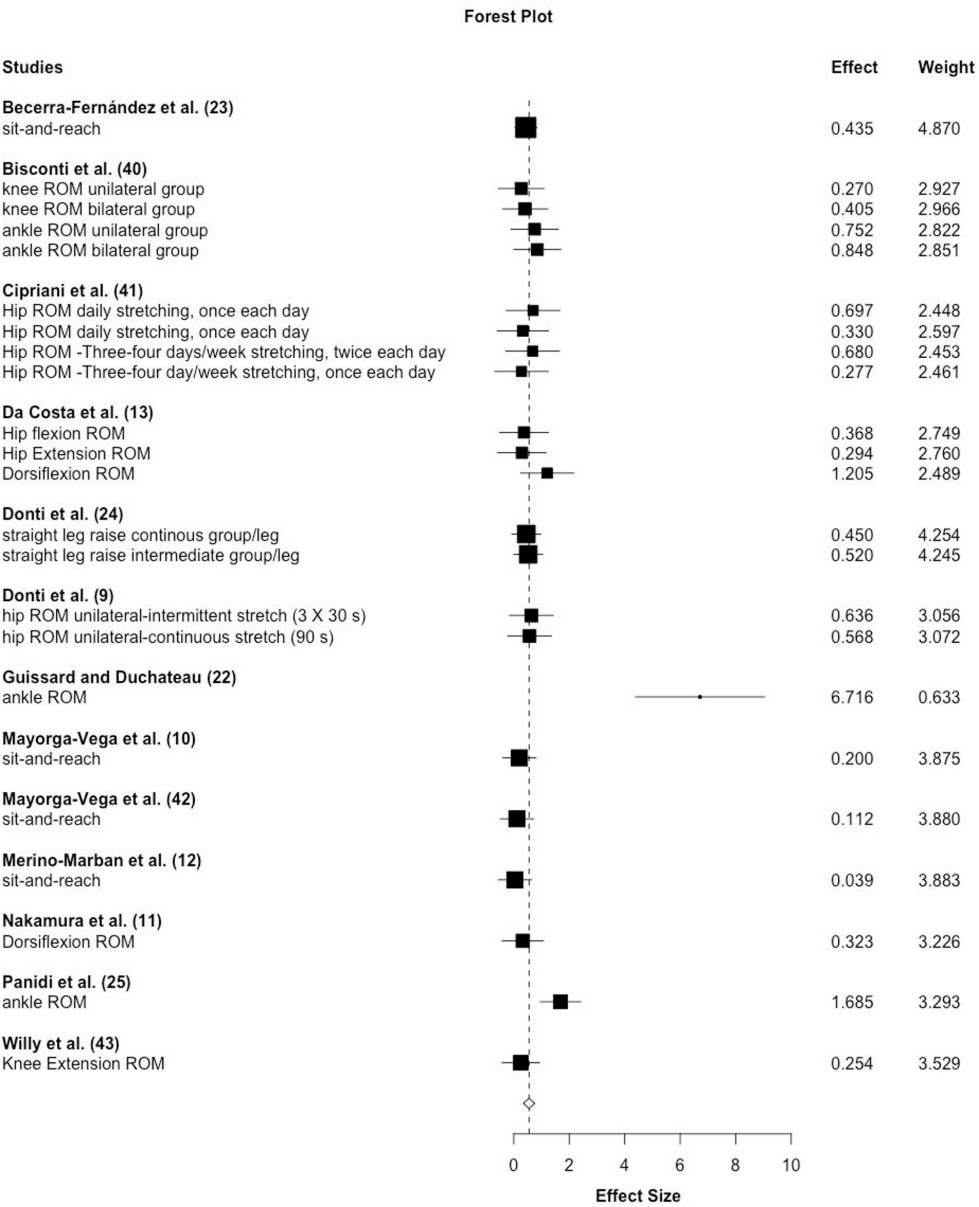
Forest plot for pre-intervention to detraining comparison of ROM changes extracted from included studies

Two to 6 weeks after the post-intervention (= detraining phase), ROM values were still elevated compared to the pre-intervention with a moderate magnitude of change (ES = 0.55; 95% CI: 0.33–0.77, *p* < 0.001, Ω^2^ = 0, τ^2^ = 0.17) (see Fig. 5). Effects were not significantly moderated by stretching duration per bout, weekly frequency or intervention period (*p* = 0.38–0.92).

### Post-Intervention to Detraining Comparison

**Fig. 6:**
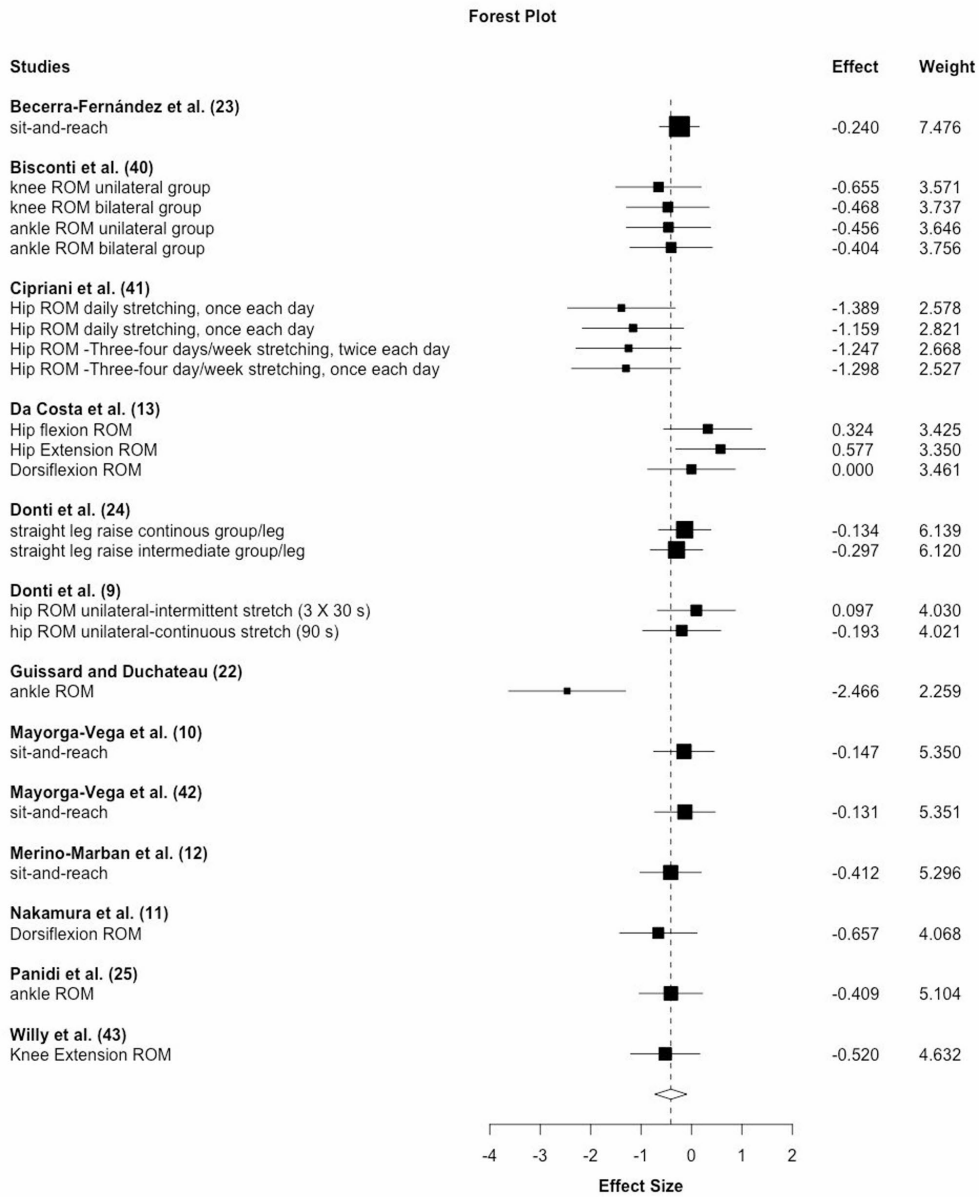
Forest plot for post-intervention to detraining comparisons of ROM changes extracted from included studies

With an ES of -0.41 (95% CI: -0.73 – -0.09), *p* = 0.016 without inter study or intra study heterogeneity (τ^2^ = 0.09 and Ω^2^ = 0) 17 effects extracted from 13 studies showed a significant reduction in flexibility during detraining. These assessments were conducted 2 to 6 weeks after the post-intervention measurement (Fig. 6). Meta regression showed that frequency per week significantly moderated the results (*p* = 0.008), while stretching duration per bout, duration of detraining or duration of the training period did not reach the level of significance (*p* = 0.11–0.55).

### Subgroup and Sensitivity Analyses

Although there was negligible outcome heterogeneity in the overall effect size pooling, methods heterogeneity could be reduced using subgroup and/or sensitivity analyses.

*Sensitivity analysis to remove outlier*

In the overall analysis, the Guissard and Duchateau [22] study showed more than doubled effect sizes compared to the remaining study effects. Therefore, a sensitivity analysis was performed excluding this outcome to improve comparability and avoid unreasonable comparisons.

Removing this study, the overall analysis effect size was reduced to ES = 0.83, 95% CI: 0.50–1.17 and *p* < 0.001 τ^2^ = 0.02 and Ω^2^ = 0.0 with a decrease in flexibility from post-intevention to detraining with ES=-0.34, 95% CI: -0.60 – -0.08, *p* = 0.02 τ^2^ = 0.02 and Ω^2^ = 0.0. Effects from pre- intervention to detraining test were therefore still significant with ES = 0.47, 95% CI: 0.3–0.64, *p* < 0.001, τ^2^ = 0.0 and Ω^2^ = 0.009). Forest plots without the study of Guissard and Duchateau [22] are shown in supplementary file 2–4 (S2-4). S2 shows pre-intervention to post-intervention, S3 pre-intervention to detraining, and S4 post-intervention to detraining.

*Sensitivity analysis for stretching type*

Most studies included in the analysis used static stretching. Only one study [23] applied ballistic stretching. Accordingly, a sensitivity analysis was performed restricting the included studies to static stretching interventions. Without affecting certainty of evidence classification, there were still significant effects (ES = 0.95, 95% CI: 0.53–1.38, *p* < 0.001) without relevant inter- and intra-study heterogeneity (Ω^2^ = 0.0, τ^2^ = 0.37) from pre to post, while small magnitude ROM decreases were observed in detraining (ranging from two to six weeks) with ES=-0.43, 95% CI: -0.80 – -0.06, *p* = 0.03 without quantifiable outcome heterogeneity (τ^2^ = 0.12 and Ω^2^ = 0). This led to a sustained moderate magnitude increase in ROM from pre-to detraining testing with an ES = 0.57 (95% CI: 0.32–0.81, τ^2^ = 0.20 and Ω^2^ = 0).

*Responder subgroup analysis*

One important concern when examining the effects of detraining on a physical fitness parameter is whether performance is improved during the training period. In the present meta-analysis, there was one study in elderly individuals [13] in which two of the ROM measures (hip flexion and hip extension, see Fig. 4) did not improve with training. Therefore, to minimize bias due to lack of improvement in the training period, we performed a subgroup analysis including the studies that showed significant effects (i.e., the 95% confidence intervals did not cross the zero line). Excluding the non-responders from pre to post increased the effect size to ES = 1.01, 95% CI: 0.62–1.4, *p* < 0.001 with trivial outcome heterogeneity (Ω^2^ = 0.0, τ^2^ = 0.30). Since only existing intervention induced ROM increases can diminish over time, also the post-test to detraining testing effect size decreased (ES=-0.48, 95% CI: -0.77 - -0.19, *p* = 0.004) without outcome heterogeneity. Therefore, after two to six weeks, compared to the pre-test, in the detraining test there were still moderate magnitude flexibility increases observable (ES = 0.55, 95% CI: 0.30–0.80, *p* < 0.001).

*Activity level during detraining phase*

Another subgroup analysis was performed separating studies where the participants were active in sports, and likely used the ROM gained during the training phase throughout the detraining period [9, 24, 25] as opposed to the remaining studies where no such activity was reported during detraining. This analysis revealed for the non-active subgroup an increase in ROM from Pre to post with ES = 0.93, 95% CI: 0.42–1.44, *p* = 0.003, opposed by the active subgroup with an ES = 0.95, − 0.47–2.38, *p* = 0.10. From post to detraining, the non-active subgroup showed a decrease with ES = − 0.52, 95% CI: − 0.97 to − 0.39, *p* = 0.04) while the active group failed in reaching the level of significance, which indicated no decrease (*p* = 0.09). Considering the pre- to detraining change an ES = 0.38 (0.15–0.6 95% CI, *p* = 0.008) and 0.93 (95% CI: 0.42–1.44, *p* = 0.003) for the non-active and active subgroup was shown, respectively. As described in previous analyses, the inter- and intrastudy heterogeneity remained non-existent to small.

**Fig. 7:**
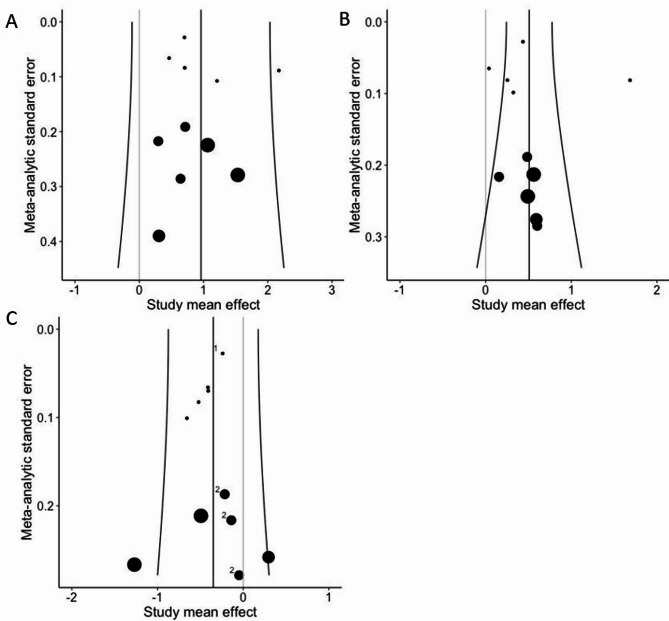
Funnel plot for visual inspection of the risk of publication bias for the pre-intervention to post-intervention (**A**), the pre-intervention to detraining (**B**) as well as the post-intervention to detraining (**C**) comparisons. Size of the dots reflect number of within study effects pooled for ES calculation

Figure 7 shows the funnel plots for the pre-intervention to post-intervention (A), the pre-intervention to detraining (B) as well as the post-intervention to detraining (C). In accordance with visual inspection, Egger's regression tests did not reach the level of significance in any of the three comparisons. (*p* = 0.05 to 0.36).

### Confidence in Cumulative Evidence

Detailed GRADE analyses can be found in supplementary file 3 (S3). In this study, eight randomized controlled trials and five controlled trials without randomization were included thus, GRADE started assuming moderate quality.

For pre- to post stretching, the quality of evidence was not downgraded for Risk of Bias, inconsistency of the results or indirectness but was downgraded by one level for publication bias (i.e., detailed but not exhaustive searching approach). Since a moderate to large effect size (ES: 0.93; 95% CI: 0.54–1.31) and a dose-response effect were found for ROM gains, the studies examining ROM gains were upgraded to high quality.

The studies examining ROM gains from pre-intervention to detraining and from post-intervention to detraining also started from a moderate quality of evidence. These studies were not downgraded for Risk of Bias, inconsistency of the results or indirectness but were downgraded by one level for publication bias (i.e., detailed but not exhaustive searching approach) and by one more level for the absence of a dose-response effect. Since moderate to low effect sizes were found for ROM gains from pre-intervention to detraining and from post-intervention to detraining (ES: 0.55; 95% CI: 0.33–0.77 and 0.41; 95% CI: − 0.73 to − 0.09, respectively), these studies were downgraded to low quality of evidence.

For ROM gains, from pre- to post intervention, the analysis showed that we can have considerable confidence that the true effect is similar to the estimated effect. However, from post-intervention to detraining and from pre-intervention to detraining the quality of the evidence was low, suggesting that our confidence in the estimate of the effect is limited, and the true effect may be substantially different from the observed estimate.

## Discussion

This systematic review and meta-analysis aimed to examine changes in ROM following stretching training and subsequent detraining. The main meta-analysis, including a total of 13 studies and 556 participants, indicated that stretching training for five to 15 weeks, induces large increases in ROM that are maintained above pre-stretching/baseline levels during detraining with a moderate effect, while small ROM decreases were observed from post-intervention until the end of the detraining period, especially in inactive participants.

Similarly to a previous meta-analysis [2] from our research group, the current analysis showed pre-intervention to post-intervention ROM increases with a large magnitude of change following long-term stretching training (5–15 weeks) (ES = 0.93, 95% CI: 0.54–1.31, *p* < 0.001). Nevertheless, the main research question to be answered with the current meta-analysis was the effect of a detraining period on ROM gains following training. Based on 13 eligible studies and 23 effect sizes, this meta-analysis indicated that ROM gains were maintained at the end of the detraining period compared with the pre-intervention level (ES = 0.55; 95% CI: 0.33–0.77, *p* < 0.001). However, ROM was slightly lower at the end of the detraining period compared with the post-intervention level (ES = − 0.41; 95% CI: − 0.73 to − 0.09), *p* = 0.016). Considering post-intervention to detraining ROM changes, similar to Thomas et al. [4] frequency of stretch training sessions per week moderates the increases in ROM. According to this evidence, the amount of stretching trainings per week seems to be an important variable in gaining ROM. However, such a moderating effect was not evident in this study for pre-intervention to post-intervention or for pre-intervention to detraining ROM. Considering static stretching only, similar results were reported (pre to post: ES = 0.95; Pre to detraining: ES = 0.57; Post to detraining: ES=-0.43). Regarding the activity levels of the participants during the detraining phase (i.e., athletes or recreationally active participants) our data have shown that ROM was maintained from post-intervention to detraining in active participants. Hence, according to our data it might be that a person whose sport involves movements across the whole ROM of the joint in everyday training has an advantage in ROM maintenance when specific stretching training is stopped. However, no difference between activity levels has been seen between pre to post or pre to detraining ROM. Moreover, by only considering responders to the stretch training (i.e., pre to post) slightly insignificant higher effect sizes were found (pre to post: ES = 1.01; Pre to detraining: ES = 0.55; Post to detraining: ES = − 0.48). Additionally, in neither of the further potential moderating variables such as stretching duration per bout, frequency per week, duration of detraining or duration of the training period we have seen a significant effect.

This study found that an average of approximately 8.7 weeks of stretching training induces large increases in ROM that are maintained above baseline levels, following on average 4.3 weeks of detraining showing that flexibility gains are maintained to some degree over time. In general, longer-term training interventions induce large ROM gains [25–27] and despite the long detraining periods observed in the included studies, ROM decreases were small. This is of great importance for both, clinical and athletic populations when planning medical therapies or training periodization.

Certainly, here the question that arises is what is the physiological mechanism for the changes in ROM following a stretch training and if the same mechanisms are responsible for the maintenance of ROM when the training is stopped? Considering the most common stretching technique, namely static stretching, it has been reported that frequent stretches for several weeks can increase ROM of a joint [2–4] and if applied with a high volume (≥ 15 min a day and 5 times a week) they can even increase muscle strength as well [28]. Additionally, besides the theory of altered pain perception [29] we have shown in recent meta-analyses that structural changes such as a decrease in muscle stiffness [30, 31] and/or increased fascicle lengths [32] may also contribute to the gains in ROM following chronic stretching. Nakamura et al. [11] reported an increased ankle ROM and decreased muscle stiffness of the gastrocnemius medialis following 5 weeks of stretch training of the plantar flexors (2 × 30 min/week), however, following the five weeks of detraining both ROM and muscle stiffness went back to baseline values. As in our current analysis, ROM values during detraining were significantly higher compared to pre-intervention values, our results were different from the study of Nakamura et al. [11] as they found no significant difference between pre-intervention and detraining ROM values. A potential explanation for the different findings might be that participants in Nakamura et al. [11] stretched for 5 weeks only, which can be considered as short compared to the average of our eligible studies in our analysis (8.7 ± 3.2 weeks). However, according to the data of Nakamura et al. [11] as well as on the detraining review on strength training [33] it can be assumed that potential structural changes in the muscle due to the stretch intervention (e.g., decreased muscle stiffness) may be reversed because of detraining. Muscle structural changes following stretching training may also be associated with ROM maintenance during detraining. However, only one study [25] examined gastrocnemius medialis and lateralis muscle morphology during the training and detraining period. Notably, in that study, changes in ankle angle, fascicle length and anatomical cross section area were maintained following the 3 weeks of detraining compared to post-intervention possibly due to the characteristics of the stretching protocol (12 weeks of long duration and high intensity stretching) and/or changes in fascicle length or tissue stiffness [25].

According to our findings, it is important to note that if flexibility is a determining factor for sports performance or daily living, detraining must be avoided and hence, a flexibility-enhancing/maintenance method must be employed. Not only stretch training can enhance flexibility [2–4] but also other methods such as full ROM resistance training [34] or foam rolling [35]. Even, compared to stretch training both foam rolling [36] and resistance training [34, 37] showed to be similarly effective for chronically increasing the ROM of a joint. Hence, it is up to the preferences of coaches, practitioners, and individuals to choose one or more methods to sustainably increase or maintain the ROM of a joint. Indeed, considering stretch training and detraining, the lowest effective dose of stretching for ROM maintenance has yet to be determined. Reid and Kim [38] reported that following stretching training for six weeks five times a week, one group reduced the stretches per week to one session while another group reduced the stretches to three sessions for another six weeks. While the group with the three sessions per week could maintain ROM, the group with one stretch session a week lost the ROM within these six weeks. Hence, the authors suggested a minimum of three stretching training sessions per week to maintain the improvements in ROM. This was confirmed by a study conducted by Rancour et al. [39], in which participants underwent daily stretching training for four weeks. One group continued stretching two to three times per week for an additional four weeks, while the other group stopped stretching altogether. Only the group that continued stretching was able to maintain the range of motion (ROM) gains achieved during the initial training period.

This research has limitations, and some questions could not be answered from the available data. A key limitation of this systematic review is the low quality of the available evidence on the detraining effects of stretching, which reduces the confidence in the reported findings and limits the ability to draw firm conclusions about the true effect. Another shortcoming is that it is not possible to estimate the length of the detraining period after which ROM decreases below the post-training level. Hence, future research should monitor ROM frequently during detraining, so that it would be possible to estimate its rate of decline after training. Additionally, it would be worth evaluating how much time it would take for the participants to return to the pre-intervention ROM levels. Furthermore, the populations taking part in the eligible studies were quite heterogeneous. The data from our meta-analysis in almost half of the included studies (see Table 1) are based on a very young population (< 18 years) and hence, care should be taken not to generalize the findings to all age levels. Additionally, while three studies had athletes as a sample [9, 24, 25], the remaining studies deal mainly with inactive persons.

However, according to our subgroup analysis, we could not find such differences. As our results are based only on a small number of effect sizes, future studies should take this into account. Another limitation was the variety of the ROM measurements used (see Table 1; Outcome), which highlights the heterogeneity of the included studies.

## Conclusion

In conclusion, the meta-analysis revealed that long-term stretch training leads to increased ROM with a large effect size, and that detraining resulted in ROM levels higher than pre-intervention but lower than post-intervention levels. This reduction was observed only in inactive participants. Therefore, we suggest that in training and rehabilitation settings, where flexibility is an important consideration, stretch training should not be discontinued, or similarly effective methods should be employed to at least maintain flexibility.

## Supplementary Information

Additional file 1.

Additional file 2.

Additional file 3.

Additional file 4.

## Data Availability

The corresponding author can provide the requested data upon request.
