# Supplementary figures and images for: Detraining Effects Following Chronic Stretching Training on Range of Motion: A Systematic Review and Meta-Analysis
Source: Sports Med Open. 2025 Nov 21;11:141. doi: 10.1186/s40798-025-00935-5 (PMC12638565; doi:10.1186/s40798-025-00935-5)

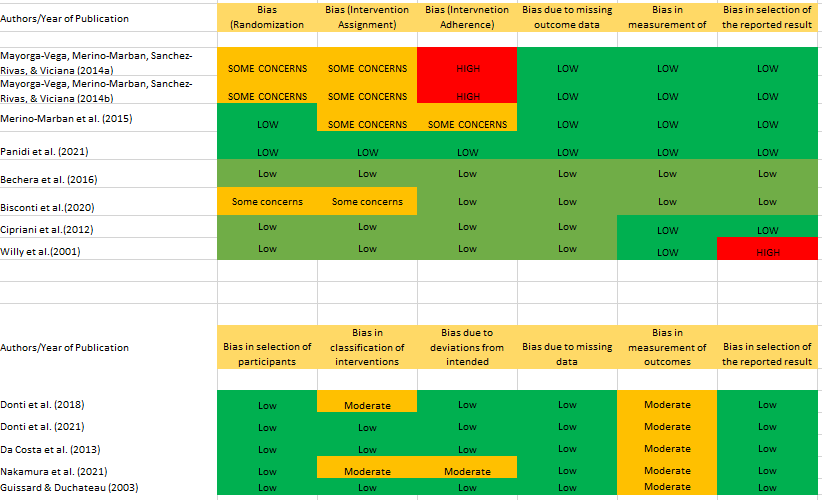

Supplement: Supplementary file 1 — Additional file 1. [file 40798_2025_935_MOESM1_ESM.png]

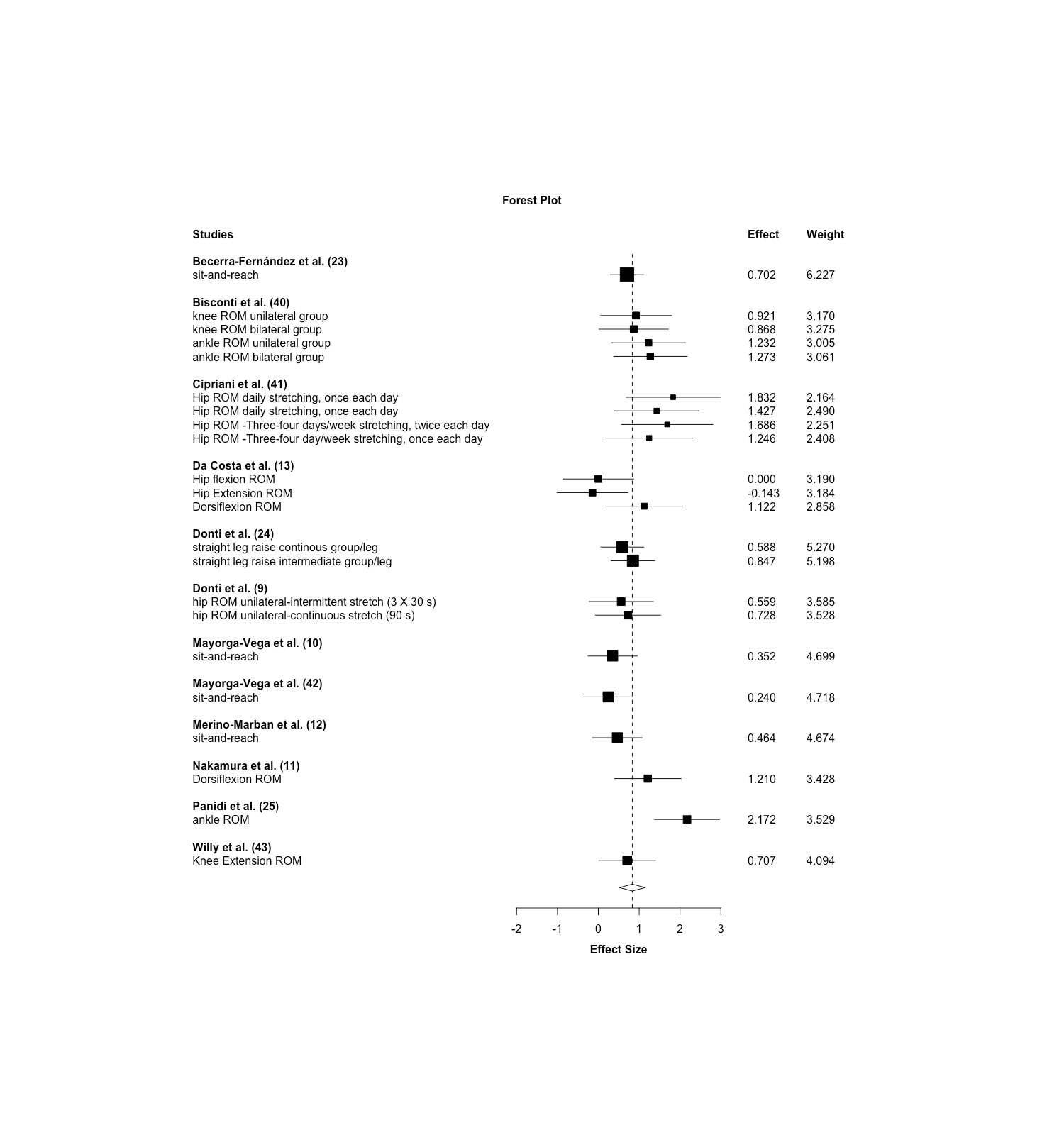

Supplement: Supplementary file 2 — Additional file 2. [file 40798_2025_935_MOESM2_ESM.png]

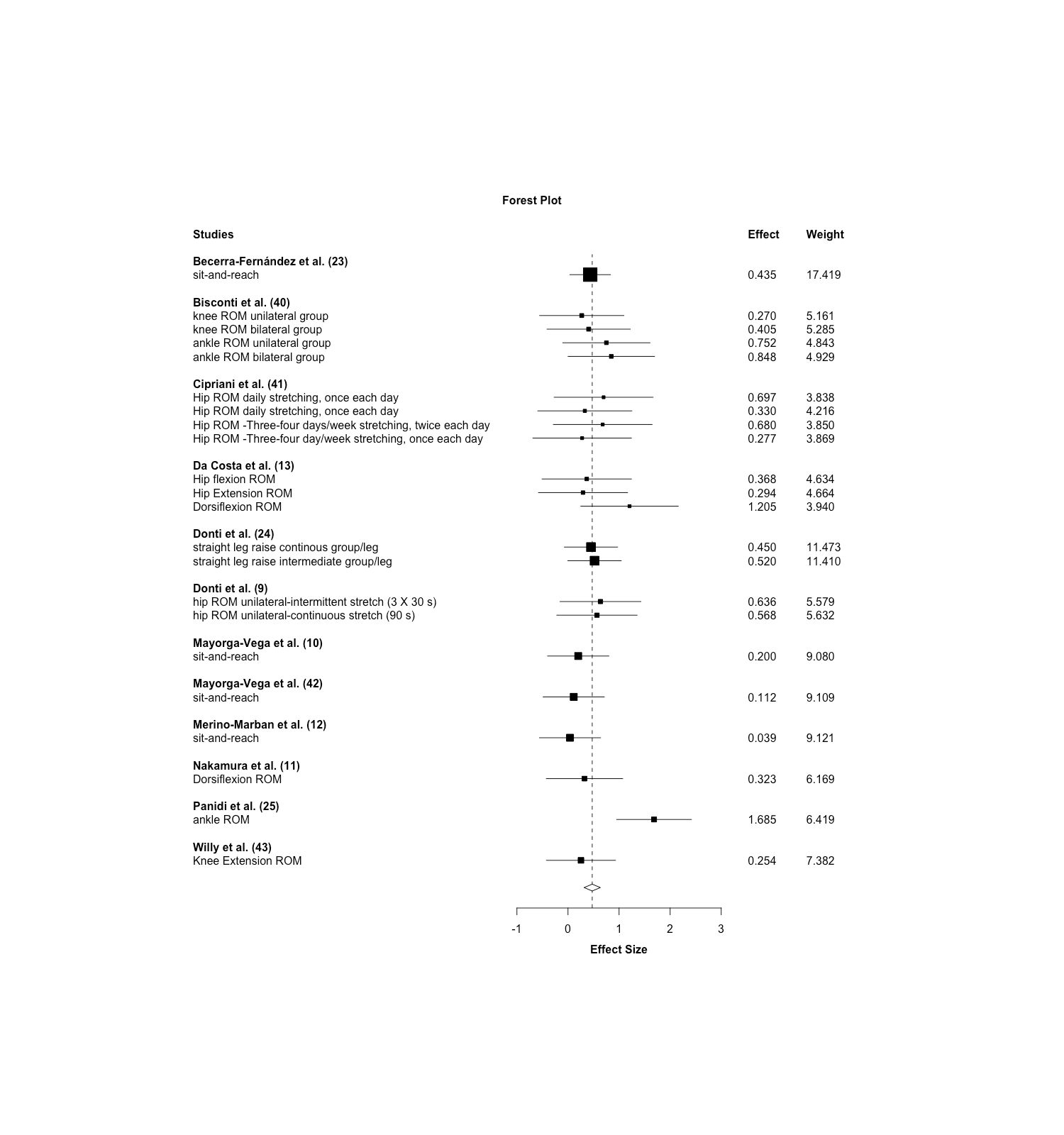

Supplement: Supplementary file 3 — Additional file 3. [file 40798_2025_935_MOESM3_ESM.png]

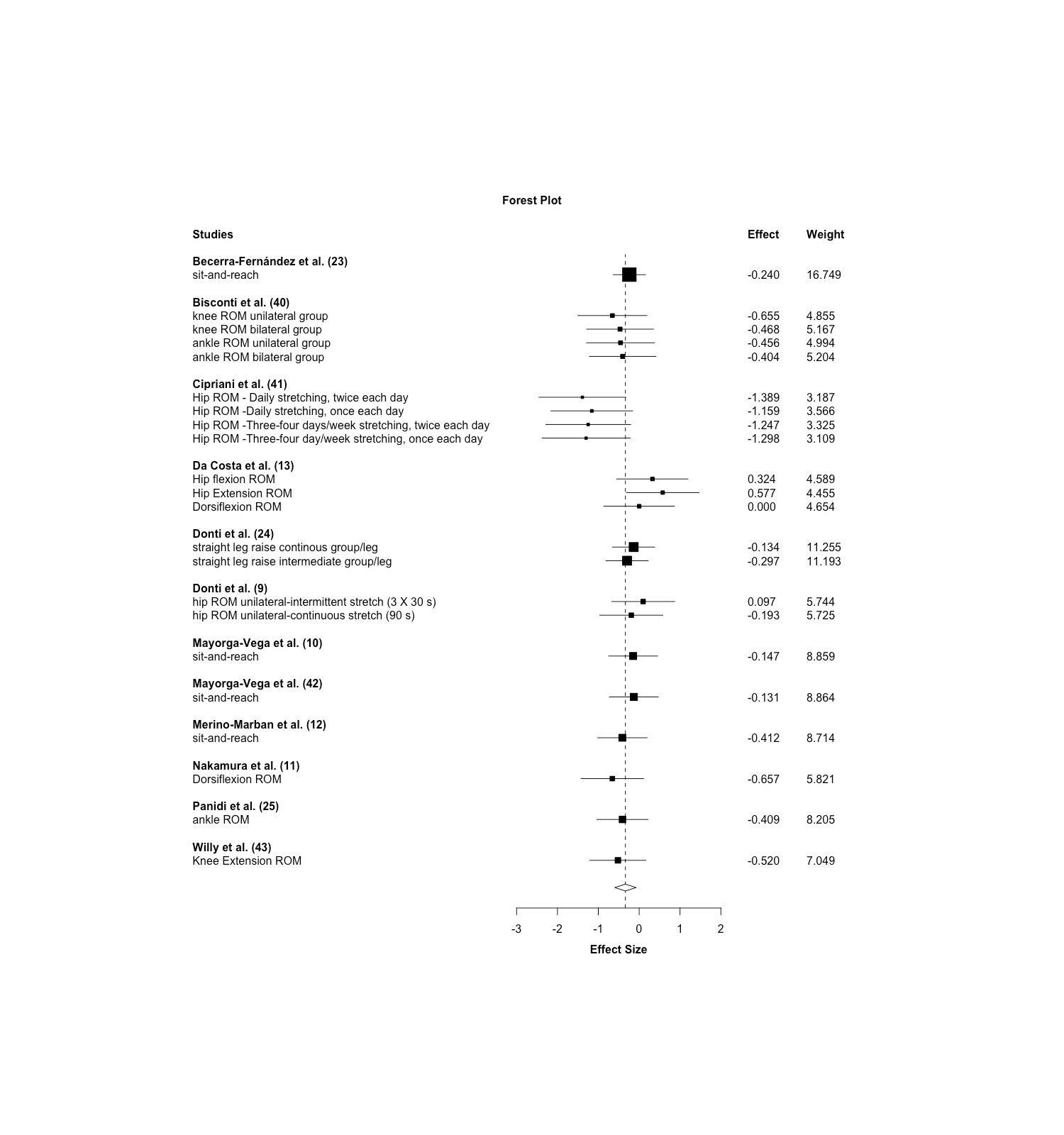

Supplement: Supplementary file 4 — Additional file 4. [file 40798_2025_935_MOESM4_ESM.png]
